# Whole-body MRI for staging and interim response monitoring in paediatric and adolescent Hodgkin’s lymphoma: a comparison with multi-modality reference standard including ^18^F-FDG-PET-CT

**DOI:** 10.1007/s00330-018-5445-8

**Published:** 2018-06-15

**Authors:** Arash Latifoltojar, Shonit Punwani, Andre Lopes, Paul D. Humphries, Maria Klusmann, Leon Jonathan Menezes, Stephen Daw, Ananth Shankar, Deena Neriman, Heather Fitzke, Laura Clifton-Hadley, Paul Smith, Stuart A. Taylor

**Affiliations:** 1 Centre for Medical Imaging, University College London, Charles Bell House, 2nd floor, 43-45 Foley Street, London, W1W 7TS UK; 2 Department of Radiology, University College London Hospitals, 235 Euston Road, London, NW1 2BU UK; 3 Cancer Research UK and UCL Cancer Trial Centre, University College London, 90 Tottenham Court Road, London, W1T 4TJ UK; 4 Institute of Nuclear Medicine, University College London and NIHR University College London Hospitals Biomedical Research Centre, 235 Euston Road, London, NW1 2BU UK; 5 Department of Paediatric Haemato-Oncology, University College London Hospitals, 235 Euston Road, London, NW1 2BU UK

**Keywords:** Whole-body scan, Diffusion-weighted MRI, Tumour staging, Treatment, Hodgkin lymphoma

## Abstract

**Objectives:**

To prospectively investigate concordance between whole-body MRI (WB-MRI) and a composite reference standard for initial staging and interim response evaluation in paediatric and adolescent Hodgkin’s lymphoma.

**Methods:**

Fifty patients (32 male, age range 6–19 years) underwent WB-MRI and standard investigations, including ^18^F-FDG-PET-CT at diagnosis and following 2–3 chemotherapy cycles. Two radiologists in consensus interpreted WB-MRI using prespecified definitions of disease positivity. A third radiologist reviewed a subset of staging WB-MRIs (*n* = 38) separately to test for interobserver agreement. A multidisciplinary team derived a primary reference standard using all available imaging/clinical investigations. Subsequently, a second multidisciplinary panel rereviewed all imaging with long-term follow-up data to derive an enhanced reference standard. Interobserver agreement for WB-MRI reads was tested using kappa statistics. Concordance for correct classification of all disease sites, true positive rate (TPR), false positive rate (FPR) and kappa for staging/response agreement were calculated for WB-MRI.

**Results:**

There was discordance for full stage in 74% (95% CI 61.9–83.9%) and 44% (32.0–56.6%) of patients against the primary and enhanced reference standards, respectively. Against the enhanced reference standard, the WB-MRI TPR, FPR and kappa were 91%, 1% and 0.93 (0.90–0.96) for nodal disease and 79%, < 1% and 0.86 (0.77–0.95) for extra-nodal disease. WB-MRI response classification was correct in 25/38 evaluable patients (66%), underestimating response in 26% (kappa 0.30, 95% CI 0.04–0.57). There was a good agreement for nodal (kappa 0.78, 95% CI 0.73–0.84) and extra-nodal staging (kappa 0.60, 95% CI 0.41–0.78) between WB-MRI reads

**Conclusions:**

WB-MRI has reasonable accuracy for nodal and extra-nodal staging but is discordant with standard imaging in a substantial minority of patients, and tends to underestimate disease response.

**Key Points:**

*• This prospective single-centre study showed discordance for full patient staging of 44% between WB-MRI and a multi-modality reference standard in paediatric and adolescent Hodgkin’s lymphoma*.

*• WB-MRI underestimates interim disease response in paediatric and adolescent Hodgkin’s lymphoma*.

*• WB-MRI shows promise in paediatric and adolescent Hodgkin’s lymphoma but currently cannot replace conventional staging pathways including* ^*18*^*F-FDG-PET-CT*.

**Electronic supplementary material:**

The online version of this article (10.1007/s00330-018-5445-8) contains supplementary material, which is available to authorized users.

## Introduction

Hodgkin’s lymphoma (HL) is the most common adolescent lymphoma [1]. Positron emission tomography computed tomography (^18^F-FDG-PET-CT) remains the first-line imaging technique [2], providing both structural and functional metabolic information to localise and characterise tumour burden. Furthermore, as a biomarker of glucose metabolism, uptake of the radiotracer ^18^F-2-fluro-2-deoxy-d-glucose (^18^F-FDG) provides a more accurate assessment of treatment response than simple structural evaluation [2–4]. ^18^F-FDG-PET-CT, however, imparts a substantial dose of ionising radiation, which may be associated with increased risk of secondary malignancies [2, 5]. This is a concern in the paediatric age group given the increased sensitivity of tissues to radiation exposure, coupled with the significant improvement in long-term survival [6, 7].

Whole-body magnetic resonance imaging (WB-MRI) is an attractive alternative to ^18^F-FDG-PET-CT as it does not impart ionising radiation and can provide high quality anatomical images through the body in less than 1 h [6, 8, 9]. Moreover, there is evidence suggesting that diffusion-weighted imaging (DWI) may act as a surrogate for the functional information provided by ^18^F-FDG [10, 11]. DWI captures water movement within tissue and its functional parameter, the apparent diffusion coefficient (ADC), is a marker of tissue cellularity and related to glucose metabolism [12, 13].

There is increasing supportive literature for implementation of WB-MRI in lymphoma staging pathways [14–18], although such data remains relatively sparse in the paediatric population [19]. Extrapolation from adult studies may be flawed given the complexities of imaging patients with smaller body habitus, the challenges of prolonged WB-MRI protocols for younger patients, and potential differences in disease patterns and behaviours between paediatric and adult patients, and within lymphoma subtypes.

The purpose of this study was to investigate prospectively the concordance between WB-MRI and a composite reference standard based on clinical evaluation, histology and standard staging imaging including ^18^F-FDG-PET-CT for initial staging and interim treatment response monitoring in paediatric and adolescent Hodgkin’s lymphoma.

## Material and methods

We conducted a prospective single-arm cohort study in a single tertiary referral centre, following ethical permission (Clinicaltrials.gov number: NCT01459224).

Consent for study investigations, including collection of anonymised patient data, was obtained from patients and or their parents/guardians according to the institutional and ethical committee guidelines.

## Patient population

Consecutive patients were prospectively identified between December 2011 and August 2014 inclusive from the paediatric lymphoma service of University College London Hospital.

Inclusion criteria were age 5–20 years (inclusive), histological confirmation of HL or clinically suspected HL (classical HL and nodular lymphocyte predominant HL) undergoing staging investigations pending final biopsy confirmation, and patients/guardian consent. All patients were either recruited to the Euronet PHL-C1 or PHL-LP1 trials [20] or were due to undergo treatment using the chemotherapy regimens of these trials.

Exclusion criteria included previous diagnosis of HL without being disease free for 5 years, previous chemotherapy and or radiotherapy within the previous 2 years, pregnancy or breastfeeding and any known contraindication to MRI.

## Summary of study conduct

All recruited patients underwent the standard staging investigations employed at the recruiting institution: (i) whole-body ^18^F-FDG-PET-CT, (ii) anatomical WB-MRI sequences with single-phase post-contrast acquisition through the upper abdomen, (iii) abdominal ultrasound in cases of equivocal solid organ involvement and (iv) contrast-enhanced chest CT (CE chest CT) scan in case of equivocal lung involvement.

To provide a comprehensive “stand-alone” WB-MRI protocol, for the purposes of the current study, the WB-MRI protocol was extended to include whole-body DWI and dynamic contrast-enhanced (DCE) sequences though the liver/spleen and chest, as well as the standard basic anatomical sequences.

Thereafter, patients underwent interim ^18^F-FDG-PET-CT (iPET-CT) within 14 days of completing the first two (Euronet PHL-C1) or three (Euronet LP1) cycles of chemotherapy for initial treatment response evaluation. Patients were invited to undergo a second WB-MRI (iWB-MRI) and were followed for a minimum 24 months post chemotherapy.

## Imaging protocols

Full descriptions of the WB-MRI and standard imaging protocols are given in the Electronic Supplementary Material (ESM). WB-MRI sequence parameters are shown in Supplemental Table 1.

## Staging imaging interpretation

A full description of WB-MRI and standard imaging interpretation is provided in the ESM. In brief, ^18^F-FDG-PET-CT was interpreted by a nuclear medicine physician (LM with more than 10 years of experience) and basic anatomical WB-MRI sequences including single-phase post-contrast sequences through the upper abdomen (but excluding DWI and DCE sequences), abdominal ultrasound and chest CT images (when available) were evaluated by consultant paediatric radiologist (PH with 11 years of experience in WB-MR imaging). WB-MRI was interpreted by two radiologists (SAT and SP with 5 and 7 years’ experience of WB-MRI) in consensus utilising all the available sequences (including the DWI and DCE images). The radiologists were blinded to the clinical history (other than the diagnosis of lymphoma) and all other investigations. A third blinded radiologist (MK with 3 years’ experience of WB-MRI) interpreted a subset of 38 WB-MRI data sets to test interobserver agreement with the primary consensus read.

The disease status for 18 nodal and 14 extra-nodal sites was evaluated, as well as the final Ann Arbor stage derived using predefined definitions based on size, ^18^F-FDG uptake and ADC (based on previous pilot data [21]; see ESM).

## Interim treatment response evaluation

Interim ^18^F-FDG-PET-CT (iPET-CT) and WB-MRI (iWB-MRI) were interpreted by the same individuals who read the initial staging investigations.

For WB-MRI, the ADC criteria for nodal response were based on those derived from previous work investigating ADC changes in responsive and non-responsive nodal disease [21] (Table 1). Extra-nodal response was evaluated by qualitative assessment of iWB-MRI classifying response into four categories: (a) locally undetectable (complete response), (b) locally detectable but reduction in size or number of deposits (partial response), (c) locally unchanged (no change in the number or size of deposits) and (d) locally progressive (increase in size or number of deposits).

**Table 1:** Nodal disease response assessment

Disease response	Definition for standard imaging tests	Definition for WB-MRI scan
Complete response (CR)	Residual tumour volume is < 25% of initial staging or ≤ 2 ml and PET negative	Residual tumour volume is < 25% of initial staging or ≤ 2 ml and ADC > 30% change compared to pretreatment value
Partial response (inadequate) (PRi)	Residual tumour volume < 75% but ≥ 50% of initial staging, or disease is PET avid (focal or diffuse uptake exceeding that of mediastinal blood pool in a location incompatible with normal anatomy or physiology)	Residual tumour volume < 75% but ≥ 50% of initial staging, or fractional change in ADC < 70% compared to pretreatment value
Partial response (adequate) (PRa)	Residual tumour volume ≤ 50% but ≥ 25% of initial staging, and all disease is PET negative (avidity not exceeding that of mediastinal blood pool)	Residual tumour volume ≤ 50% but ≥ 25% of initial staging, and fractional change in ADC ≥ 70% compared to pretreatment value
No change (NC)	Residual tumour volume ≥ 75% but < 125% of initial staging	Residual tumour volume ≥ 75% but < 125% of initial staging
Progression (PRO)	Residual tumour volume ≥ 125%	Residual tumour volume ≥ 125%

*WB-MRI* whole-body MRI, *PET* positron emission tomography, *ADC* apparent diffusion coefficient

A full description of interim response evaluation is provided in ESM.

## Primary and enhanced reference standards

A full description is provided in ESM. In brief, the primary reference standard was assigned by a multidisciplinary team (MDT) on the basis of their assessment of all standard imaging tests together with all clinical information, including available histology.

**Fig. 1:**
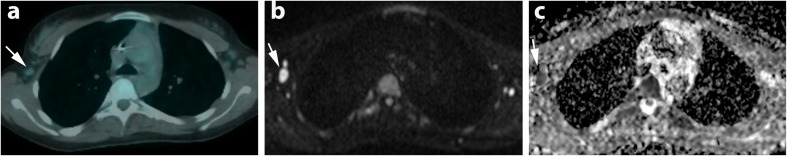
Example of ^18^F-FDG-PET-CT perceptual error. Right axillary nodal station was called negative on (**a**) ^18^F-FDG-PET-CT and positive on WB-MRI. b_500_ diffusion-weighted MRI (**b**) and apparent diffusion coefficient map (**c**) showing restricted diffusion (ADC 1.0 × 10^−3^ mm^2^/s). On retrospective evaluation of nodal station, with full follow-up data available, the expert panel judged the right axillary node (arrows) to be a positive nodal site based on ^18^F-FDG uptake, and thus a perceptual error on initial ^18^F-FDG-PET-CT interpretation

Given the potential limitations of standard imaging in staging HL, which may weaken the primary reference standard, a retrospective enhanced reference standard was also produced by a central expert panel comprising two radiologists, two nuclear medicine physicians and two paediatric haemato-oncologists. The central panel reviewed all staging, interim and end of treatment scans as well as follow-up imaging and clinical outcomes up to 24 months post chemotherapy. The panel corrected simple labelling (boundary) discrepancies that were due to differences in disease site description between tests, and thereafter any perceptual or technical failures in the primary reference standard (Fig. 1). WB-MRI perceptual errors were also noted.

## Data analysis and study power

The primary endpoint was based on achieving full (100%) concordance between WB-MRI and the primary reference standard in terms of correct disease classification for each and every anatomical site (i.e. the 18 nodal and 14 extra-nodal sites). A binary classification of each disease status as either negative or positive/equivocal was made as part of the reference standard.

See ESM for the power calculation of the study sample size.

The primary endpoint was summarised in terms of frequency and percentage of patients who had a concordance below 100% for all disease sites combined, and separately for nodal and extra-nodal sites. The median and interquartile range (IQR) discordance rate for each patient was also calculated.

The true positive rate (TPR) (sensitivity) and false positive rate (FPR) of WB-MRI were calculated for nodal and extra-nodal disease sites, along with the kappa statistic. Agreement for Ann Arbor staging and classification of interim treatment response evaluation (for positive/equivocal disease sites that were concordant at initial staging) were summarised in terms of frequency, percentages and kappa.

Sensitivity analysis were performed using the outcomes from the central review process, and the enhanced reference standard.

Specifically, the agreement analyses for staging WB-MRI were repeated:

- After correcting for anatomical boundary labelling description discrepancies only
- Against the enhanced reference standard (including correction of boundary labelling description discrepancies)
- Against the enhanced reference standard after removal of WB-MRI perceptual errors

Ann Arbor staging agreement was also assessed after integrating the results of enhanced reference standard and after WB-MRI correction for perceptual errors.

Interobserver agreement between consensus WB-MRI read and the third radiologist was tested using kappa statistics.

Statistical analysis was performed using the Stata software package (Version 14. Stata Corporation LP, College Station, Texas).

## Results

### Patient characteristics

**Fig. 2:**
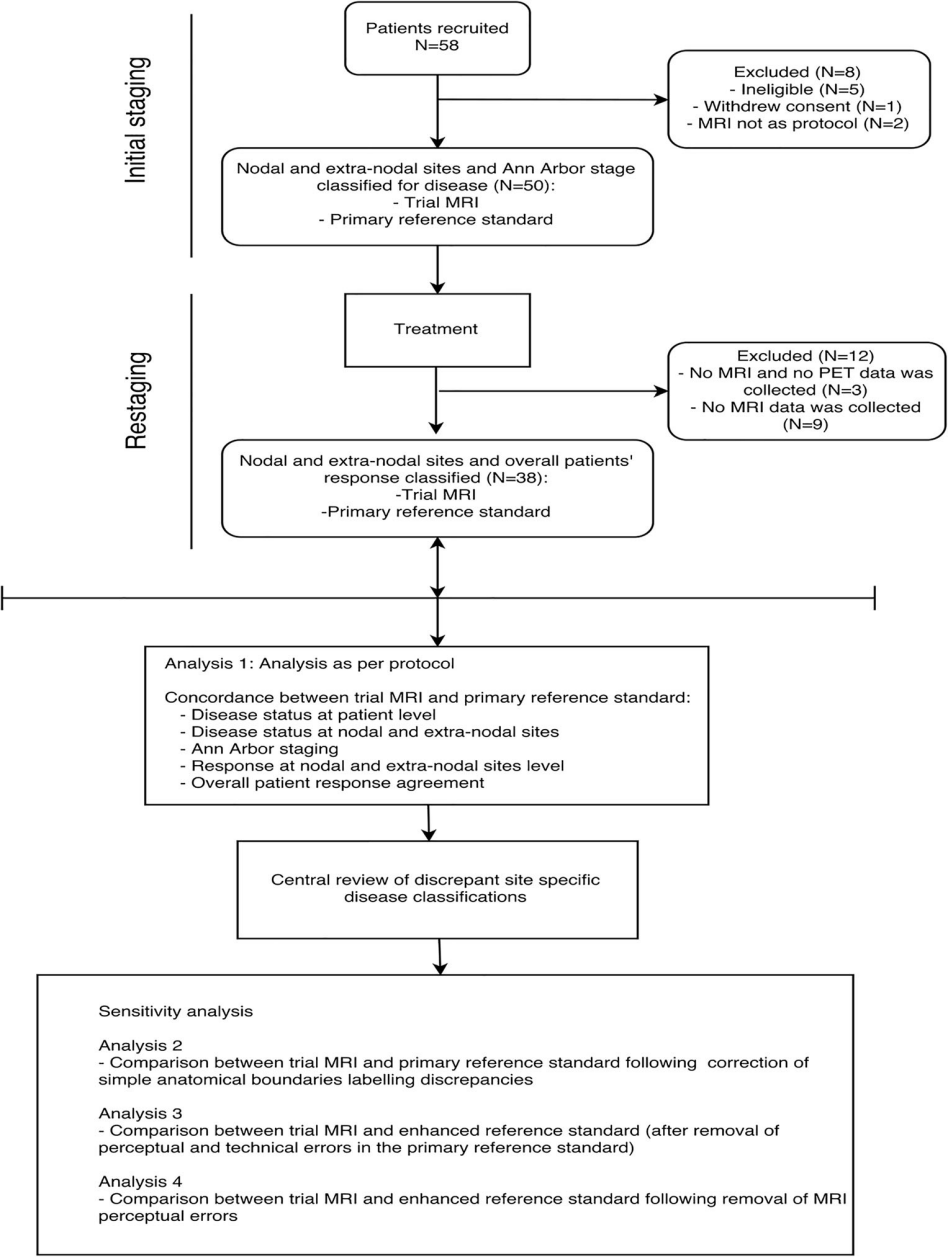
Study flowchart

Fifty-eight patients were recruited (M/F 39:19, median age 16, range 5–19 years). The study flowchart is presented in Fig. 2. Eight patients were excluded. The demographics, disease subtype and treatment regimen of the final 50 patient study cohort are shown in Table 2. Staging WB-MRI was performed within a median 2 days (range 0–20 days) of ^18^F-FDG-PET-CT without any complication, and before treatment in all patients.

**Table 2:** Patients’ cohort demographics

Baseline characteristics	*N* (%)
	*n* = 50
Age (years)	
Median (range)	16 (6–19)
Sex	
Female	18 (36%)
Male	32 (64%)
Hodgkin’s lymphoma subtype	
Classical	42 (84%)
Nodular lymphocyte predominant	8 (16%)
Chemotherapy	
OEPA	9 (18%)
OEPA/COPDAC	32 (64%)
CVP	7 (14%)
DHAP/OEPA/COPDAC	1 (2%)
Others^a^	1 (2%)

*OEPA* vincristine, etoposide, prednisolone, doxorubicin; *COPDAC* cyclophosphamide, vincristine, prednisolone, dacarbazine; *CVP* cyclophosphamide, vincristine, prednisolone; *DHAP* dexamethasone, cytarabine, cisplatin

^a^One patient with stage I and single lymph node involvement that was excised for histopathology did not received any treatment

### Central review and enhanced reference standard

Across the cohort there were 1527 disease sites [875 nodal (850 predefined sites and 25 “other” sites and 652 extra-nodal sites (650 predefined sites and 2 “other” sites)] evaluated by both WB-MRI and standard imaging.

The central review identified and resolved 44 anatomical boundary labelling description discrepancies. There were 10 nodal and 4 extra-nodal perceptual errors in the primary reference standard, together with 1 technical error.

There were 20 WB-MRI perceptual errors.

### Initial staging agreement: per patient

**Fig. 3:**
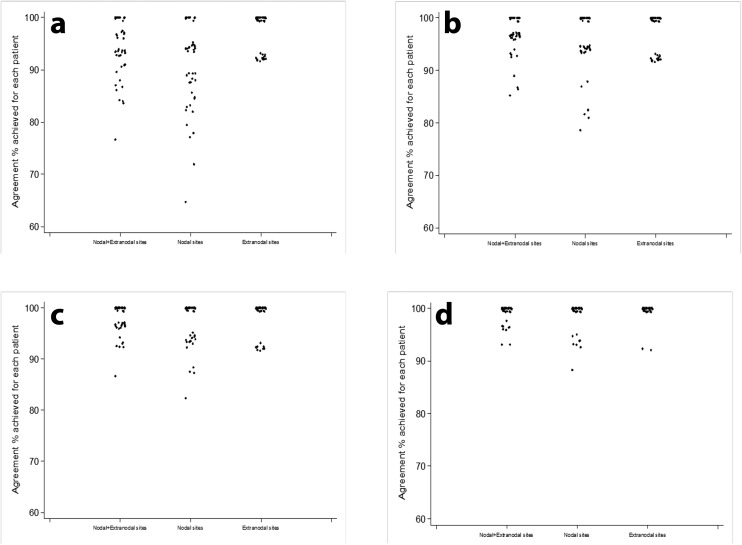
Per patient concordance rate. Concordance rate for nodal, extra-nodal and combined nodal/extra-nodal sites between **a** WB-MRI and primary reference standard prior to the removal of simple boundary classification labelling discrepancies and **b** following the removal of simple boundary classification labelling discrepancies. **c** WB-MRI and the enhanced reference standard (following removal of ^18^F-FDG-PET-CT perceptual and technical errors) and **d** WB-MRI and the enhanced reference standard following removal of WB-MRI perceptual errors. Median and interquartile range (IQR) are presented for each analysis tier

Per patient concordance rate for each analysis is shown in Table 3 and Fig. 3.

**Table 3:** Per patient concordance rate for each analysis

Concordance rate	Overall (Nodal and extra-nodal sites)	Nodal sites	Extra-nodal sites
	*N* = 50	*N* = 50	*N* = 50
	*n* (%)	*n* (%)	*n* (%)
Analysis 1^a^			
≤ 60%	–	–	–
> 60% to ≤ 80%	1 (2%)	5 (10%)	–
> 80% to ≤ 90%	8 (16%)	15 (30%)	–
> 90% to < 100%	28 (56%)	16 (32%)	14 (28%)
100%	13 (26%)	14 (28%)	36 (72%)
Analysis 2^b^			
≤ 60%	–	–	–
> 60% to ≤ 80%	–	1 (2%)	–
> 80% to ≤ 90%	4 (8%)	5 (10%)	–
> 90% to < 100%	26 (52%)	16 (32%)	14 (28%)
100%	20 (40%)	28 (56%)	36 (72%)
Sensitivity analysis 1^c^			
≤ 60%	–	–	–
> 60% to ≤ 80%	–	–	–
> 80% to ≤ 90%	1 (2%)	4 (8%)	–
> 90% to < 100%	21 (42%)	13 (26%)	9 (18%)
100%	28 (56%)	33 (66%)	41 (82%)
Sensitivity analysis 2^d^			
≤ 60%	–	–	–
> 60% to ≤ 80%	–	–	–
> 80% to ≤ 90%	–	1 (2%)	–
> 90% to < 100%	9 (18%)	7 (14%)	2 (4%)
100%	41 (82%)	42 (84%)	48 (96%)

^a^Comparison between WB-MRI and primary reference standard before correction of simple anatomical boundaries labelling discrepancies

^b^Comparison between WB-MRI and primary reference standard following correction of simple anatomical boundaries labelling discrepancies

^c^Comparison between WB-MRI and enhanced reference standard (after removal of perceptual and technical errors in the primary reference standard)

^d^Comparison between WB-MRI and enhanced reference standard following removal of WB-MRI perceptual errors

After correcting for labelling discrepancies, the discordance rate was 44% (90% CI exact 32.0–56.6%) for nodal sites and 28% (90% CI exact 17.8–40.3%) for extra-nodal sites.

Against the enhanced reference standard, the equivalent discordance rates fell to 44% (90% CI exact 32.0–56.6%) for all sites, 34% (90% CI exact 23.0–46.5%) for nodal sites and 18% (90% CI exact 9.7–29.3%) for extra-nodal sites. After removal of WB-MRI perceptual errors, the discordance rates for all, nodal and extra-nodal sites were 18% (90% CI exact 9.7–29.3%), 16% (90% CI exact 8.2–27.0%) and 4% (90% CI exact 0.7–12.1%), respectively.

### Initial staging agreement: disease site

Absolute agreement rate, TPR, FPR and Cohen’s kappa statistic for nodal and extra-nodal disease sites for each analysis are shown in Table 4.

**Table 4:** Overall true positive rate, false positive rate, agreement rate and kappa for nodal and extra-nodal staging

Analyses	Agreement rate	TPR	FPR	Kappa (95% CI)
Analysis 1^a^				
Nodal sites	91% (799/875)	81% (184/226)	5% (34/649)	0.77 (0.72–0.82)
Extra-nodal sites	98% (638/652)	72% (28/39)	< 1% (3/613)	0.79 (0.68–0.90)
Analysis 2^b^				
Nodal sites	96% (799/831)	90% (184/204)	2% (12/627)	0.89 (0.86–0.93)
Extra-nodal sites	98% (638/652)	72% (28/39)	< 1% (3/613)	0.79 (0.68–0.90)
Sensitivity analysis 1^c^				
Nodal sites	97% (809/831)	91% (192/210)	1% (4/621)	0.93 (0.90–0.96)
Extra-nodal sites	99% (643/652)	79% (30/38)	< 1% (1/614)	0.86 (0.77–0.95)
Sensitivity analysis 2^d^				
Nodal sites	99% (822/831)	97% (203/210)	< 1% (2/621)	0.97 (0.95–0.99)
Extra-nodal sites	> 99% (650/652)	95% (36/38)	0% (0/616)	0.97 (0.93–1.00)

*TPR* true positive rate, *FPR* false positive rate, *CI* confidence interval

^a^Comparison between WB-MRI and primary reference standard before correction of simple anatomical boundaries labelling discrepancies

^b^Comparison between WB-MRI and primary reference standard following correction of simple anatomical boundaries labelling discrepancies

^c^Comparison between WB-MRI and enhanced reference standard (after removal of perceptual and technical errors in the primary reference standard)

^d^Comparison between WB-MRI and enhanced reference standard following removal of WB-MRI perceptual errors

Against the enhanced reference standard, the WB-MRI TPR, FPR and kappa agreement were 91%, 1% and 0.93 (95% CI 0.90–0.96) for nodal disease and 79%, < 1% and 0.86 (95% CI 0.77–0.95) for extra-nodal disease.

Following removal of WB-MRI perceptual errors, the TPR, FPR and kappa agreement were 97%, < 1% and 0.97 (95% CI 0.95–0.99) for nodal and 95%, 0% and 0.97 (95% CI 0.93–1.00) for extra-nodal assessment compared to enhanced reference standard. There were seven WB-MRI false negative nodal sites due to technical failures (i.e. not visible in retrospect), two false positive nodal sites and two false negative extra-nodal sites (Supplemental Table 4).

### Ann Arbor staging agreement

Based on enhanced reference standard, there were 2, 26, 5, 14 and 3 patients with Ann Arbor stage 1, 2, 3, 4 and 4E, respectively.

Agreement between WB-MRI and the primary reference standard was substantial (kappa 0.66, 95% CI 0.50–0.83) with staging concordant in 39/50 (78%) patients (Supplemental Table 5).

**Fig. 4:**
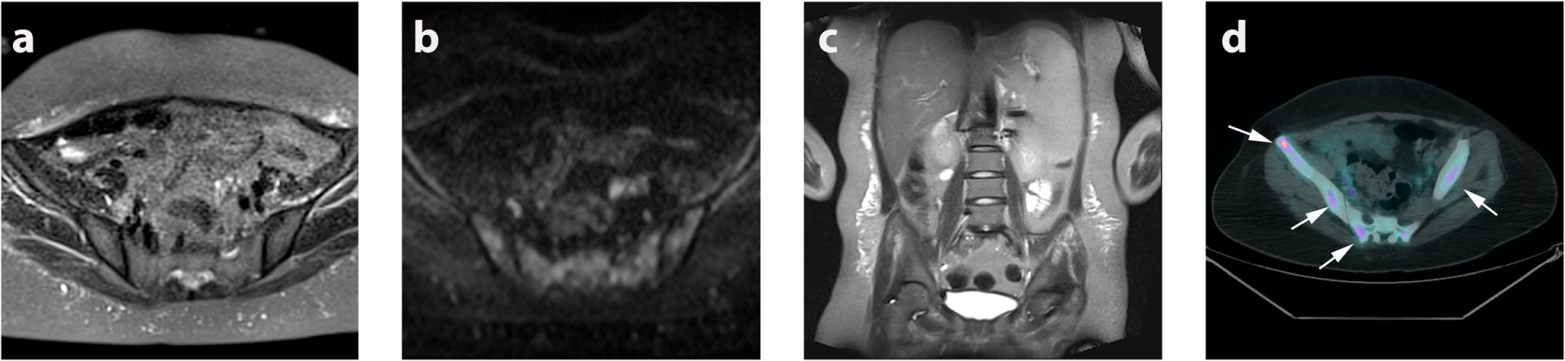
Example of WB-MRI technical error. False negative WB-MRI technical error resulting in under-staging of a 15-year-old female patient with multifocal bone marrow involvement; **a** axial STIR-HASTE, **b** DWI b_500_ and **c** coronal STIR-HASTE MRI show no discernible bone marrow abnormality. **d** ^18^F-FDG-PET-CT, however, demonstrates multifocal bone marrow metastasis (arrows)

Prior to removal of WB-MRI perceptual errors, agreement between WB-MRI and the enhanced reference was substantial (kappa 0.72, 95% CI 0.56–0.88) with concordance in 41/50 (82%) patients. After removal of the WB-MRI perceptual errors concordance was achieved in 48/50 patients (96%), (kappa 0.94, 95% CI 0.85–1.00). Two patients were under-staged as a result of technical failure of WB-MRI compared to enhanced reference (Fig. 4 and Supplemental Table 5).

### Interim treatment response agreement

Thirty-eight of the 50 patients were evaluable for interim treatment response analysis (Fig. 2). iWB-MRI scans were acquired within a median 1 day (range 0–7 days) of iPET scans.

On a per patient basis, iWB-MRI agreed with the primary reference standard response classification in 25/38 patients (66%, 6 PR and 19 CR), underestimating response in 10 (26%) patients and overestimating response in 3 (8%) patients (kappa 0.30, 95% CI 0.04–0.57) (Table 5).

**Table 5:** Per patient interim treatment response for whole-body MRI compared to combined reference standard

Overall patient response		Combined reference			
		CR (*n*)	PR (*n*)	NC (*n*)	PRO (*n*)
Trial WB-MRI	CR (*n*)	19	2	0	0
	PR (*n*)	9	6	1	0
	NC (*n*)	1	0	0	0
	PRO (*n*)	0	0	0	0

*CR* complete response, *n* number, *NC* no change, *PR* partial response, *PRO* progression, *WB-MRI* whole-body MRI

There were 143 nodal and 26 extra-nodal positive concordant sites evaluable for interim treatment assessment.

iWB-MRI agreed with the primary reference standard response classification in 126/143 (88%) nodal sites, underestimating response in 3 (2%) sites and overestimating response in 14 (10%) (Supplemental Table 6).

**Fig. 5:**
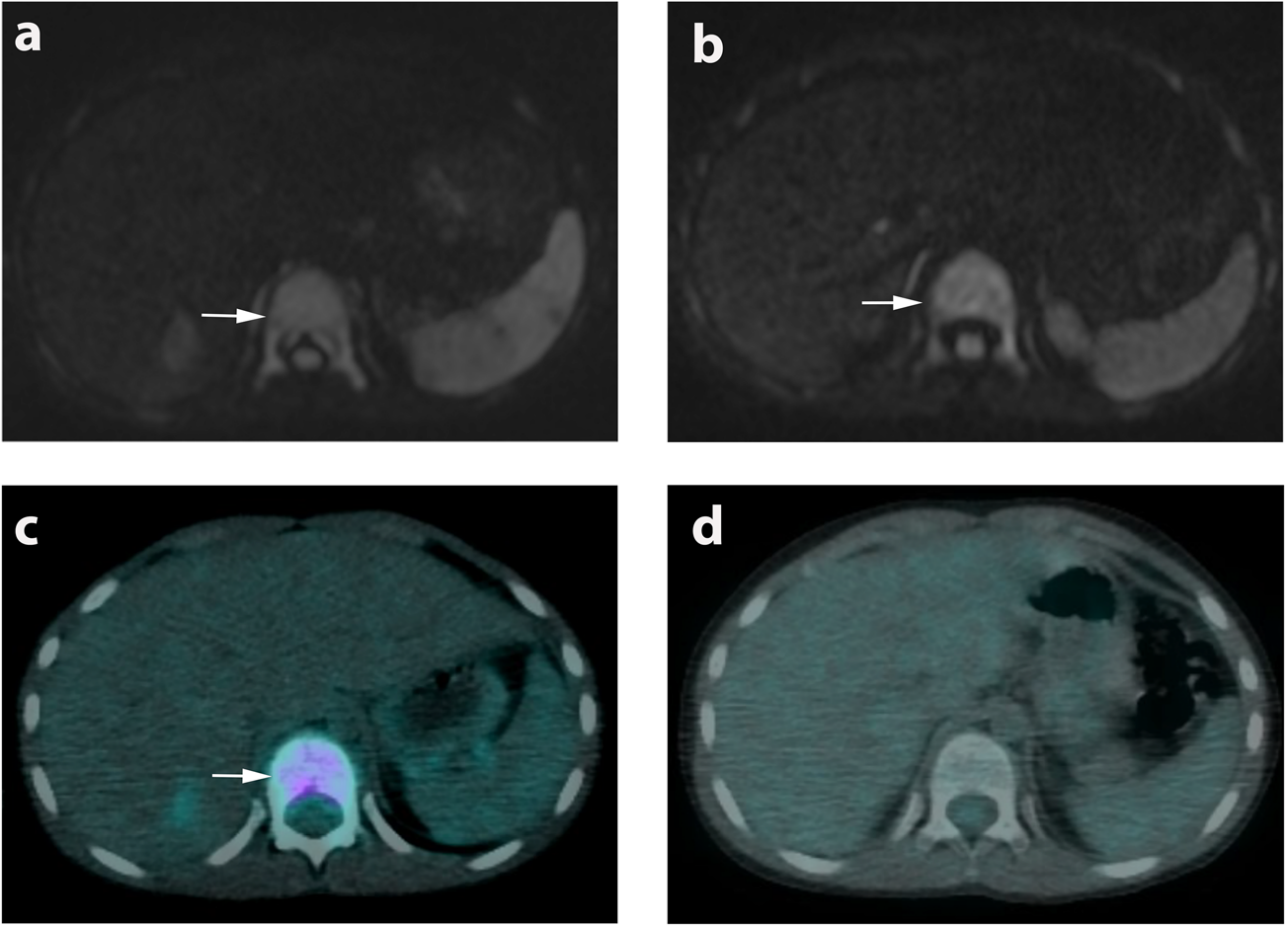
Example of discrepant interim treatment response classification. WB-MRI and ^18^F-FDG-PET-CT of an 8-year-old male subject with Ann Arbor stage 4 disease. Baseline WB-MRI (**a**) and ^18^F-FDG-PET-CT (**c**) showing involvement of entire T11 vertebrae (arrows). Interim WB-MRI (**b**) showing no signal intensity changes (arrow) whilst interim ^18^F-FDG-PET-CT (**d**) demonstrated complete response. Patient remained in remission following chemotherapy

iWB-MRI agreed with primary reference standard response classification in 17/26 (66%) of extra-nodal sites. In the remaining 9 (34%) sites, WB-MRI underestimated response (Fig. 5). Specifically, WB-MRI underestimated bone marrow response in four patients (three with reduced but persistent detectable disease and one with unchanged disease), and for spleen and lung in two and three patients respectively (all five with reduced but persistent disease on WB-MRI). All nine sites showed complete response on primary reference standard.

### WB-MRI interobserver agreement

There was a good agreement between the consensus WB-MRI and the 3rd radiologist reads for nodal (kappa 0.78, 95% CI 0.73–0.84), extra-nodal staging (kappa 0.60, 95% CI 0.41–0.78) and Ann Arbor staging (kappa 0.62, 95% CI 0.32–0.73).

## Discussion

In the current study we compared WB-MRI with a combined multi-modality reference standard based mainly on standard imaging (notably ^18^F-FDG-PET-CT) but including clinical and histological data for staging and interim treatment response monitoring in paediatric HL.

Overall, we found that WB-MRI has reasonable accuracy for nodal and extra-nodal staging but did not achieve full concordance for all disease sites in a substantial minority of patients, and tends to underestimate disease response.

Our findings of intrinsically high sensitivity and specificity for nodal and extra-nodal staging confirm the data of Littooij et al. who performed a similar staging study in a cohort of 33 paediatric patients with a range of lymphoma phenotypes [19], and mirror those of Mayerhoefer et al. [17] who studied a cohort of 140 adult patients. In line with previous work [14], we utilised a rigorous consensus review process taking into consideration all long-term imaging and clinical follow-up to create an enhanced reference standard, thereby correcting deficiencies in standard staging pathways, and providing a more realistic evaluation of the accuracy of WB-MRI. Against this enhanced reference, WB-MRI, sensitivity for extra-nodal disease was still modest at 79%. We also retrospectively corrected WB-MRI perceptual errors to indicate the theoretical “best” technical performance of WB-MRI, which increased nodal sensitivity to 97% and extra-nodal disease sensitivity to 95%. Clearly perceptual errors are unavoidable so such corrected data will overestimate the performance of WB-MRI, but particular emphasis should be made on detecting extra-nodal disease during radiologist training.

Our primary analysis, and one rarely performed in the literature, is how often WB-MRI achieved full concordance with standard imaging for each and every disease site in an individual patient. Such data is clinically highly relevant, as patients with early unfavourable response will often undergo targeted radiotherapy to individual involved nodal stations following chemotherapy [22]. Against the enhanced reference standard, full concordance for nodal disease was achieved in 66% of patients, which increased to 84% after removal of WB-MRI perceptual errors. Although such data is encouraging, there is a substantial minority of patients with discordant findings to standard staging, which may have treatment implications. Our data suggests that using ADC as a surrogate for ^18^F-FDG uptake, although promising [12, 21], is currently insufficient. It is clear there is overlap in ADC between malignant lymph nodes and normal/reactive lymph nodes and the optimal ADC cut-off remains unclear, and requires further investigation [23].

Although access to new ^18^F-FDG-PET-MR technology is currently very limited, this platform my ultimately prove to be the investigation of choice and prospective studies are currently underway [24].

The accuracy of iWB-MRI for interim treatment response assessment is under investigation, but far from proven [11–13, 18, 25].

Using simple visual inspection of DWI images, Mayerhoefer et al. [18] reported that region-based agreement between WB-DWI with ^18^F-FDG-PET-CT was 99.2% after 1–3 therapy cycles in their cohort of 51 adult patients with various lymphoma types, and Tsuji et al. [11] found that WB-DWI was concordant with ^18^F-FDG-PET-CT in 100% of cases (*n* = 19) with lesion negative interim scans.

One potential advantage of applying quantitative ADC cut-offs for response assessment is to improve the specificity of simple visual assessment. Littooij et al. [13], for example, reported that applying an ADC cut-off value of 1.21 × 10^−3^ mm^2^/s increased specificity for residual nodal disease detection by nearly 30% compared to visual inspection only.

By applying a similar ADC cut-off, we found that iWB-MRI agreed with the reference standard in a moderate 66% of patients.

One particular observation was the persistence of abnormal DWI bone marrow signal after successful treatment, resulting in underestimation of response by MRI and highlighting a limitation of visual response of extra-nodal disease on DWI. Quantitative ADC measurements my aid the differentiation between persistent tumour and treatment necrosis [26] and requires further investigation. For example, post-chemotherapy ADC monitoring in multiple myeloma has already shown promise for response assessment [27]. Such evidence is currently lacking in paediatric lymphoma, although intuitively ADC assessment could also be beneficial, and requires further evaluation.

Our study has some limitations. Our standard staging protocol, although primarily based on ^18^F-FDG-PET-CT, also includes anatomical MRI sequences. There is a theoretical risk of incorporation bias as these sequences were available to the MDT when they created the primary reference standard [28]. However, DWI and DCE sequences were not available to the MDT, and the complete WB-MRI examination was viewed as a standalone examination by radiologists blinded to all other clinical information. As noted, ^18^F-FDG-PET-CT is the mainstay of staging at our institution. Any incorporation bias would favour WB-MRI and the fact we report modest WB-MRI performance data suggests that any bias did not influence the overall study outcome.

We used an unblinded expert panel opinion and long-term follow-up data to derive the enhanced reference standard, an approach commonly used in studies of imaging diagnostic accuracy in absence of a single reference standard [14, 15].

We have used the highest *b* value of 500 s/mm^2^ for DWI disease assessment. We acknowledge that a higher *b* value between 800 and 1000 s/mm^2^ would have been in line with current recommendations on WB-DWI [29]. However, our ADC cut-off parameters were derived from previous pilot work [21] using similar DWI protocol as the current study. It is, however, possible that using a higher *b* value of 800–1000 s/mm^2^ instead of 500 s/mm^2^ could improve disease detection because of a superior lesion-to-contrast ratio. This could, for example, potentially decrease perceptual errors for extra-nodal disease assessment.

We used both qualitative and quantitative MRI assessment for staging and response monitoring. The generalizability of ADC quantitation across institutions and platforms, however, remains challenging [30, 31]. We also used a consensus reading paradigm for WB-MRI as at the time of the study set-up this mirrored our usual clinical practice and the use of ADC cut-offs was deemed exploratory [32]. We did reassuringly demonstrate good interobserver agreement with a third radiologist (as have others [19]). However, given that consensus reading is not widely used, it cannot be assumed that our data is representative of standard clinical practice where single reading is more common.

It has been shown that quantitative ADC changes following chemotherapy may differ between HL and non-HL subtypes of lymphoma [25] and our data is applicable to paediatric and adolescent HL.

Finally, although ADC changes as early as 1 week post chemotherapy have been documented for very early response assessment in adult lymphoma [33], the delayed second time point for iWB-MRI in our study was based on institutional guidelines for iPET-CT, Euronet trial [20] and recommendations in the literature [34, 35]. It would now be useful to investigate whether WB-MRI performs better for response assessment if performed at an earlier time point (e.g. 2 weeks) after chemotherapy.

In conclusion, WB-MRI with DWI has reasonable intrinsic diagnostic performance for nodal and extra-nodal staging of paediatric HL. However, in a substantial minority of patients it fails to achieve full concordance with standard imaging for all disease sites. WB-MRI has reasonable accuracy for interim treatment response classification but tends to underestimate disease response, particularly in extra-nodal disease sites. Overall, although promising, WB-MRI with DWI cannot currently replace standard imaging investigations in paediatric and adolescent Hodgkin’s lymphoma and further research is required, particularly to derive optimum ADC cut-offs for disease status, and the significance of persistent extra-nodal abnormality following treatment.

## Electronic supplementary material

ESM 1 (DOC 343 kb)

## Abbreviations

ADC: Apparent diffusion coefficient
DWI: Diffusion-weighted imaging
FPR: False positive rate
HL: Hodgkin’s lymphoma
IQR: Interquartile range
MDT: Multidisciplinary team
TPR: True positive rate
WB-MRI: Whole-body MRI
