# Supplementary material for: Whole-body MRI for staging and interim response monitoring in paediatric and adolescent Hodgkin’s lymphoma: a comparison with multi-modality reference standard including 18F-FDG-PET-CT
Source: Eur Radiol. 2018 Jun 15;29(1):202–12. doi: 10.1007/s00330-018-5445-8 (PMC6291431; doi:10.1007/s00330-018-5445-8)
Supplement: Supplementary file 1 — (DOC 343 kb) [file 330_2018_5445_MOESM1_ESM.doc]

### **Whole body MRI protocol**

Patients were imaged on a 1.5T MRI system (Avanto, Siemens, Erlangen, Germany) from the skull to mid-thigh in the supine position, using the manufacturer’s body and spine array coils. Immediately prior to imaging, 0.3 mg/kg of body weight of hyoscine butylbromide (Buscopan; Boehringer Ingelheim, Ingelheim, Germany) was administered.

At the recruitment site, basic anatomical sequences are routinely acquired to supplement the low dose CT component of the ^18^F-FDG PET-CT protocol. In brief, respiratory and electrocardiographically gated axial and coronal whole body fat suppressed T2-weighted MRI together with axial periodically rotated overlapping lines with enhanced reconstruction (PROPELLER) were acquired through the chest (in maximum inspiration) and complemented by a single-phase post-contrast T1-weighted sequence (3D Fast low angle shot technique, FLASH) through upper abdomen at 25 seconds following gadolinium injection.

As part of the trial intervention, and to provide a comprehensive “stand alone” protocol, the WB-MRI scan was extended and the above sequences were complemented by the addition of axial free-breathing DWI (with four b-values b**_0_**, b**_100_**, b**_300_** and b**_500_**) acquired through the whole-body, together with multiphase breath-hold dynamic contrast enhanced T1-weighted series through the liver and spleen (following a single intravenous dose of 0.1 mmol/kg body weight of gadoterate meglumine (Dotarem; Laboratoire Guerbet, Aulnay-sous-Bois, France) as described previously [1]. Axial and coronal breath-hold post-contrast T1-weighted MRI of the lungs were then acquired following the liver and splenic acquisition. ADC maps were generated using vendor’s software.

The full WB-MRI scanning protocol was completed within 60 minutes. WB-MRI scanning parameters are summarised in supplemental Table 1.

### **^18^F-FDG PET-CT protocol**

^18^F-FDG PET-CT scans were performed with integrated PET/CT scanners (Discovery ST or Discovery VCT, GE Healthcare, Waukesha Wisconsin, USA). Patients fasted for 6 hours and blood glucose levels were tested to exclude hyperglycemia (levels >180mg/dL). For pediatric patients, the doses were adjusted according to the European Association of Nuclear Medicine (EANM) pediatric dosage card [2]. ^18^F-FDG (14MBq - 370MBq) was intravenously injected 60 minutes before imaging. Prior to acquiring the whole-body PET 3D emission scan, a non-contrast CT was obtained for attenuation correction (80-120 kVp, modulated mA [10-200mA], pitch 1.375, 3.75mm slice thickness). Images were acquired at 3 min per bed position as per departmental pediatric protocol.

**Contrast-enhanced chest CT**

CE chest CT was performed in cases of equivocal lung findings on ^18^F-FDG PET-CT [3], after intravenous contrast administration (2.0ml/kg Omnipaque 300, General Electric Healthcare, Milwaukee, Wisconsin, USA), using a 64-multi-detector row CT scanner (Siemens Somatom 64, Siemens, Erlangen, Germany) (120kVp, 45 ref. mAs, 0.5s rotation time, 64x0.6mm detectors, pitch 1.4, 24x1.2 collimation).

### **Abdominal ultrasound**

Abdominal ultrasound was performed by a consultant radiologist in case of equivocal solid organ involvement on other cross-sectional imaging.

### **Standard staging imaging interpretation**

^18^F-FDG PET-CT was interpreted by a nuclear medicine physician (Blinded for review) on a dedicated workstation (Xeleris 2; GE Healthcare, Milwaukee, Wisconsin, USA). The basic anatomical WB-MRI sequences including single-phase post-contrast sequences through the upper abdomen (but excluding DWI and dynamic contrast enhanced sequences), abdominal ultrasound and high-resolution contrast-enhanced chest CT images (when available) were evaluated by consultant pediatric radiologist (Blinded for review) using a standard picture archiving and communication system (PACS) (IMPAX version 6.5.1; Agfa-Gevaert, Morstel, Belgium). The readers derived the disease status for the 18 nodal and 14 extra nodal, as well as the final Ann Arbor stage. The 18 nodal disease sites were cervical [right (R) and left (L)], supraclavicular (R and L), subpectoral (R and L), axillary (R and L), mediastinal, splenic hilar, liver hilar, mesenteric, retroperitoneal, iliac (R and L), inguinal (R and L) and “other” sites, and 14 extra-nodal disease sites were lung (R and L), pleura, pericardium, chest wall, liver, spleen, kidney (R and L), stomach, bowel, pancreas, bone marrow and “other” sites.

Definitions of nodal disease positivity were those utilized by the Euronet PHL-C1 or LP1 trials [4], and based on long-axis size and ^18^F-FDG uptake in comparison to background activity (supplemental Table 2).

Disease volume was derived using all three-axis measurements [(X x Y x Z)/2] for largest nodal mass in each nodal site, or for the conglomerate nodal mass if no discrete nodal tissue was visible, and was used for subsequent treatment response evaluation.

The criteria for extra-nodal disease status using standard imaging is summarised in supplemental Table 3.

### **Staging Whole body MRI interpretation**

Two radiologists (Blinded for review) in consensus reviewed anonymized WB-MRI datasets as a “stand alone staging investigation” using Osirix (Version 4.0, Apple, California, USA) viewing software, utilizing all the available sequences (including the DWI and dynamic contrast enhanced images). The radiologists were blinded to the clinical history (other than the diagnosis of lymphoma) and all other investigations.

The disease status for the same 18 nodal disease sites and 14 extra-nodal disease sites, as well as final Ann Arbor stage were derived according to predefined trial criteria. Specifically for lymph nodes, disease positivity was defined using a combination of size and ADC criteria (supplemental Table 2). Size criteria for disease positivity were based on those used by the Euronet PHL-C1 or LP1 trials [4].

The largest diameter of a single lymph node or a lymph node conglomerate was measured on T2-weighted MRI. Nodal disease volume was derived as described above for standard imaging.

ADC quantitation was performed by placing a region of interest in the largest cross section of the node on the ADC map, guided by anatomically matched axial fat-suppressed T2-weighted MRI. The derived ADC cut offs for nodal positivity were based on previous work [5].

The criteria for extra-nodal disease on WB-MRI was based on structural observations and MRI signal changes (supplemental Table 3).

The disease status for the same 18 nodal and 14 extra-nodal disease sites and Ann Arbor stage were derived according to predefined trial criteria.

### **Standard Imaging interim response interpretation**

The nuclear medicine physician and consultant radiologist who evaluated the standard initial staging imaging interpreted the iPET-CT and anatomical iWB-MRI sequences, and derived the treatment response for all nodal and extra-nodal disease sites using predefined trial criteria based on tumour volume and ^18^F-FDG uptake (used by the Euronet trials, Table 1) [4]. Consistent with the initial staging measurements, nodal response was assessed for largest diameter of a single lymph node (when visible) or a conglomerate nodal mass (if no discreet node was visible).

Extra-nodal response was classified into four categories: [a] locally undetectable (complete response), [b] locally detectable but reduction in size or number of deposits (partial response), [c] locally unchanged (no change in the number or size of deposits) and [d] locally progressive (increase in size or number of deposits).

The overall interim per patient response for standard imaging was defined using the least responsive nodal and/or extra-nodal disease sites.

### **Whole body MRI interim response interpretation**

The same radiologists who interpreted the initial staging WB-MRI, evaluated the complete iWB-MRI and derived the treatment response for all nodal and extra-nodal disease sites, again blinded to all other investigations, and using predefined criteria based on changes in nodal volume (used by the Euronet trials) [4] and ADC measurement (Table 1).

The ADC criteria for response were based on those derived from previous work [5]. Extra-nodal response was evaluated by qualitative assessment of iWB-MRI and classified into four categories as for the standard imaging described above.

The overall per patient interim response for iWB-MRI was defined using the least responsive nodal and/or extra-nodal disease sites.

## Primary reference standard

The primary reference standard for all 32 disease sites, Ann Arbor stage at initial staging, and the interim treatment response evaluation was assigned by a multi-disciplinary team (MDT) meeting attended by a consultant radiologist, nuclear medicine physician, two pediatric haemato-oncologists and a haematopathologist. The panel based their assessment on all standard imaging test results (interpreted as described above), together with all clinical information including clinical examination findings, blood test results and available histology.

**Central review of imaging discrepancies and creation of an enhanced reference standard**

Given the potential limitations of standard imaging in staging HL, and the risk of radiologist/ nuclear medicine physician perceptual errors adversely influencing the primary reference standard, a retrospective enhanced reference standard was produced to better evaluate the potential accuracy of WB-MRI. Specifically, all discrepancies between WB-MRI and standard imaging tests (including ^18^F-FDG PET-CT) at initial staging were reviewed by an expert panel comprising two radiologists (one of whom was not involved in the main trial radiological interpretation), two nuclear medicine physicians (one of whom was not involved in the main trial PET scan interpretation) and two pediatric hemato-oncologists. The panel reviewed all staging, interim and end of treatment scans and had access to follow up imaging and clinical outcomes up to 24 months post chemotherapy.

***Anatomical boundary description discrepancies***

Initially the panel corrected simple labeling discrepancies that were due to differences in disease site description between those interpreting the WB-MRI and those interpreting standard imaging, usually between adjacent anatomical sites. For example, if an involved node was described as “cervical” on WB-MRI but “supraclavicular” on standard imaging, the panel opined if this was a true discrepancy or if both modalities had described the same disease, and just classified its anatomical location differently, in which case the discrepancy was re-classified as concordant.

***Correction of perceptual and technical errors in the primary standard reference and creation of an enhanced reference standard***

Once simple anatomical boundary labeling discrepancies were corrected, the panel reviewed the remaining discrepancies and decided if the primary reference standard needed correcting based on all the available imaging and follow up data. Initially simple perceptual errors in standard imaging interpretation were corrected, for example unequivocal areas of disease positivity that responded to treatment, but were missed on the original ^18^F-FDG PET-CT interpretation and visible on the ^18^F-FDG PET-CT in retrospect on review (Fig. 1).

Thereafter positive findings on WB-MRI not visible on the standard imaging even in retrospect were reviewed to see if any were technical failures of standard imaging. Only unequivocal disease sites with clear response to treatment on WB-MRI were considered technical failures of standard imaging by the panel, otherwise such findings were classified as WB-MRI false positives. In a similar fashion, the panel identified any false positive findings on standard imaging. The creation of this enhanced reference standard aimed to define the true disease status of the patients as far as possible.

***Correction of MRI for perceptual errors***

Finally, all the WB-MRI errors against the enhanced reference standard were classified into perceptual errors when the abnormality was visible in retrospect on the WB-MRI, or technical error when it was not. By identifying and correcting MRI perceptual errors it was then possible to assess the theoretical best performance of the WB-MRI protocol.

**Sample size calculation**

Assuming a discordant rate of 20% (extrapolated from pilot data [6]), a total of 55 patients would be sufficient to exclude a discordance rate of greater than 35% with 80% power and one-sided 5% significance level. A sample size of 55 patients would also be sufficient to form a 95%CI with 20% precision around an assumed kappa of 0.86 and an assumed MRI sensitivity of at least 85% for site-specific disease [6].

**Supplemental Table 1:** Whole-body MRI sequence parameters

|  | **Axial**  **STIR HASTE** | **Coronal STIR HASTE** | **Axial STIR DWI (b0,100,300,500)** | **Axial T2 PROPELLER** | **Axial post-contrast** | **Coronal post-contrast** | **3D FLASH for DCE** |
| --- | --- | --- | --- | --- | --- | --- | --- |
|  |  |  |  | **(chest)** | **(lung)** | **(lung)** | **(liver & spleen)** |
| **TR/TE (ms)** | *800/60* | *800/60* | *4900/66* | *3000/133* | *2.85/0.99* | *2.94/1.04* | *2.87/0.93* |
|  |  |  |  |  |  |  |  |
| **Inversion time (ms)** | *130* | *180* | *180* | *N/A* | *N/A* | *N/A* | *N/A* |
|  |  |  |  |  |  |  |  |
| **Matrix** | *256×192* | *128×96* | *128×96* | *256×256* | *256×88* | *256×128* | *256×176* |
|  |  |  |  |  |  |  |  |
| **Slice Thickness (mm)** | *7* | *4* | *4* | *3* | *2.5* | *3.5* | *2.5* |
|  |  |  |  |  |  |  |  |
| **No. of slices** | *19* | *27* | *27* | *23* | *104* | *56* | *80* |
|  |  |  |  |  |  |  |  |
| **Averages** | *2* | *8* | *8* | *1* | *1* | *1* | *1* |
|  |  |  |  |  |  |  |  |
| **Echo train** | *256* | *1* | *1* | *50* | *1* | *1* | *1* |
|  |  |  |  |  |  |  |  |
| **PAT** | *2* | *2* | *2* | *1* | *1* | *1* | *2* |
|  |  |  |  |  |  |  |  |
| **Flip angle** | *180* | *90* | *90* | *150* | *15* | *15* | *9* |
|  |  |  |  |  |  |  |  |
| **Pixel spacing** | *1.56×1.56* | *0.8×0.8* | *0.8×0.8* | *1.25×1.25* | *1.4×1.4* | *1.2×1.2* | *1.56×1.56* |
|  |  |  |  |  |  |  |  |

**TR**: Repetition time

**TE:** Echo time

**PAT**: Parallel acquisition technique

**STIR:** Short tau inversion recovery

**HASTE:** Half-Fourier single shot turbo spin echo

**DWI**: Diffusion weighted imaging

**FLASH**: Fast low angle shot technique

**DCE**: Dynamic contrast enhanced

**PROPELLER**: Periodically rotated overlapping lines with enhanced reconstruction

**Supplemental Table 2:** Pre-defined criteria for nodal assessment

|  | **Standard Imaging** | | **WB-MR Imaging** |
| --- | --- | --- | --- |
|  | **Cross sectional imaging (Anatomical MRI sequences, CT component of PET-CT)** | **PET-CT Imaging *** |  |
| *Positive* | Nodes > 2cm ** | N/A | Nodes > 2cm** |
|  | Nodes 1-2 cm | FDG-PET positive | Nodes 1-2 cm with ADC ≤ 1.2 |
|  | Nodes < 1 cm | FDG-PET positive | Nodes < 1 cm with ADC ≤ 0.8 |
| *Equivocal* |  |  |  |
|  | Nodes 1-2 cm | FDG-PET equivocal | Nodes 1-2 cm with ADC >1.2 and <1.8 |
| *Negative* |  |  |  |
|  | Nodes 1-2 cm | FDG-PET negative | Nodes 1-2 cm with ADC ≥ 1.8 |
|  | Nodes < 1 cm | FDG-PET negative | Nodes < 1 cm with ADC ≥ 0.8 |

**PET**: Positron emission tomography

**FDG**: ^18^F-2-fluro-2-deoxy-D-glucose

**ADC**: Apparent diffusion coefficient

**WB-MRI**: Whole-body MRI

**DWI:** Diffusion weighted imaging

***** Involvement defined as uptake above surrounding background in a location incompatible with normal physiological activity

******Long axis diameter

The largest diameter of all nodes was measured in 3 planes (axial long and short axis and coronal cranio-caudal axis) using the fat-suppressed T2-weighted images and CT scan. Disease volume was derived using all three axis measurements [(X x Y x Z)/2] for subsequent treatment response evaluation.

**Supplemental Table 3:** Pre-defined criteria for extra-nodal assessment

| **Sites** | **Standard Imaging (Anatomical MRI sequences, PET-CT, contrast enhanced CT Chest and abdominal ultrasound)** | **WB-MRI** |
| --- | --- | --- |
| Pleura | Involvement of the pleura is assumed if    • the lymphoma is contiguous with the pleura without fat lamella  or  • the lymphoma invades the chest wall or  • a pleural effusion occurs which cannot be explained by a venous congestion.  Extension: Abnormal nodular tissue within the pleura contiguous with the main nodal mass. | Extension: Abnormal nodular moderate- high signal of equal intensity to nodal tissue within the pleura contiguous with the main nodal mass.  Separate: Abnormal high signal of fluid intensity anatomically in keeping with a pleural effusion which cannot be explained by associated pulmonary oedema; or, pleural nodules discrete to the main lymph node mass. |
| Pericardium | Pericardial involvement is assumed if  • the lymphoma has a broad area of close contact towards the heart surface beyond the valve level (ventriculus area) or  • a pericardial effusion occurs/ nodules without associated mediastinal lymph node mass.  Extension: Extensive contact between mediastinal lymph node mass and pericardium to the level of the ventricles in the presence of a pericardial effusion and / or pericardial nodules. | Extension: Extensive contact between mediastinal lymph node mass and pericardium to the level of the ventricles in the presence of a pericardial effusion and / or pericardial nodules.  Separate: Pericardial effusion / nodules without associated mediastinal lymph node mass. |
| Chest wall | Chest wall infiltration is defined as extension of a mediastinal mass on CT and/or PET positive focal chest wall lesion. | Extension: Moderate-high signal infiltration of the chest wall in continuum with a lymphatic mass/or positive focal chest wall lesion on T2-STIR, DWI and/or post-contrast. |
| Lung | A disseminated lung involvement (implying stage IV) is assumed if  • there are more than three foci or  • an intrapulmonary focus has a  diameter of more than 10 mm.  Extension: Abnormal infiltration of the lung in continuum with a lymphatic mass. | Extension: Abnormal moderate-high signal infiltration of the lung in continuum with a lymphatic mass.  Separate: Abnormal moderate-high signal focus (>1cm diameter) within the lung  discrete to lymphatic tissue or more than three foci. |
| Bone marrow | Bone involvement is assumed if a bone biopsy is positive **or** CT bony window is positive with or without further confirmation by other imaging methods in the same region **or** a positive bone scan is confirmed by either FDG-PET or MRI. | Homogenous moderate-high signal foci within bone on T2-STIR, DWI and/or post-contrast at a site discrete to the bone marrow biopsy. |
| Liver | Focal changes in the liver structure on ultrasonography that are suspicious of tumour are considered positive – independent of the FDG-PET result.  In case of doubtful involvement of liver (e.g. structures atypical of tumour in sonography or MRI) the liver is considered involved if FDG-PET is positive.  Extension: Moderate-high signal infiltration of the liver in continuum with an adjacent lymphatic mass | Extension: Moderate-high signal infiltration of the liver in continuum with an adjacent lymphatic mass on T2-STIR, DWI and/or post-contrast.  Separate: Low signal (relative to surrounding liver) discrete foci within the liver not in continuation with an adjacent lymphatic mass. |
| Spleen | Focal changes in the splenic structure on ultrasonography that are suspicious of tumour are considered positive – independent of the FDG-PET result.  In case of doubtful involvement of liver (e.g. structures atypical of tumour in sonography or MRI) the liver is considered involved if FDG-PET is positive.  Moderate-high signal infiltration of the spleen in continuum with an adjacent lymphatic mass. | Extension: Moderate-high signal infiltration of the spleen in continuum with an adjacent lymphatic mass.  Separate: Low signal (relative to surrounding spleen) discrete foci within the spleen on T2-STIR, DWI and/or DCE not in continuation with an adjacent lymphatic mass. |
| Kidney | Diffuse enlargement with distortion of the renal parenchyma **or** focal lesion on CT/MRI/US **or** PET/CT positive disease. | Global or focal renal enlargement and / or discrete renal mass. |
| Stomach | Focal thickening on CT/MRI that also demonstrates PET/CT positivity. | Marked wall thickening in a distended stomach with moderate-high signal. |
| Pancreas | Diffuse enlargement with distortion of the pancreatic parenchyma **or** focal lesion on CT/MRI/US **or** PET/CT positive disease. | Focal signal change within the pancreas or global pancreatic enlargement. |
| Bowel | Focal thickening on CT/MRI that also demonstrates PET/CT positivity. | Focal bowel wall thickening and elevated STIR-HASTE signal intensity. |

**Supplemental Table 4:** list of nodal and extra-nodal discrepancies between WB-MRI and the final enhanced reference standard (ERS) following removal of WB-MRI perceptual errors

| ***Site*** | ***WB-MRI*** | ***ERS*** | ***Reason*** |
| --- | --- | --- | --- |
| Supraclavicular (n) | *Negative* | *Positive* | ADC measurement of equivocal node by size criteria |
| Axillary (n) | *Positive* | *Negative* | ADC measurement of equivocal node by size criteria |
| Cervical (n) | *Negative* | *Positive* | Subcentimeter LN positive on FDG PET |
|
| Axillary (n) | *Negative* | *Positive* | Subcentimeter LN positive on FDG PET |
|
| Cervical (n) | *Negative* | *Positive* | Subcentimeter LN positive on FDG PET |
|
| Bone marrow (e) | *Negative* | *Positive* | Multi-focal bone marrow involvement missed on WB-MRI |
| Cervical (n) | *Positive* | *Negative* | ADC measurement of equivocal node by size criteria |
| Axillary (n) | *Negative* | *Positive* | ADC measurement of equivocal node by size criteria |
| Lung (e) | *Negative* | *Positive* | Multiple small lung foci detected on CE chest CT scan and missed on WB-MRI |
| Liver Hilar (n) | *Negative* | *Positive* | ADC measurement of equivocal node by size criteria |
| Supraclavicular (n) | *Negative* | *Positive* | Subcentimeter LN positive on FDG PET |
|

**n:** Nodal site**, e:** Extra-nodal site, **LN:** Lymph node, **ERS:** Enhanced reference standard, **PET**: Positron emission tomography

**FDG**: ^18^F-2-fluro-2-deoxy-D-glucose, **ADC**: Apparent diffusion coefficient, **CE:** Contrast enhanced, **WB-MRI:** Whole body MRI

**Supplemental Table 5:** Ann Arbor staging agreement

| **Ann Arbor Staging** | | **Primary Reference Standard** | | | | | | | | | |
| --- | --- | --- | --- | --- | --- | --- | --- | --- | --- | --- | --- |
|  |  | I | II | II E | | III | | IV | | IV E | |
|  |  | N=2 | N=28 | N=0 | | N=4 | | N=13 | | N=3 | |
| **WB MRI**  **(Analysis 1)** | **I** | 2 (100%) | 1 (4%) | - | | - | | - | | - | |
|  | **II** | - | 22 (79%) | - | | 3 (75%) | | - | | - | |
|  | **II E** | - | - | 0 (0%) | | - | | - | | 1 (33%) | |
|  | **III** | - | 4 (14%) | - | | 1 (25%) | | 1 (8%) | | - | |
|  | **IV** | - | 1 (4%) | - | | - | | 12 (92%) | | - | |
|  | **IV E** | - | - | - | | - | | - | | 2 (67%) | |
|  | | **Enhanced Reference Standard** | | | | | | | | | |
|  |  | I | II | II E | | III | | IV | | IV E | |
|  |  | N=2 | N=26 | N=0 | | N=5 | | N=14 | | N=3 | |
| **WB MRI**  **(Analysis 2)** | **I** | 2 (100%) | 1 (4%) | - | | - | | - | | - | |
|  | **II** | - | 22 (85%) | - | | 3 (60%) | | - | | - | |
|  | **II E** | - | - | 0 (0%) | | - | | - | | 1 (33%) | |
|  | **III** | - | 2 (7%) | - | | 2 (40%) | | 1 (7%) | | - | |
|  | **IV** | - | 1 (4%) | - | | - | | 13 (93%) | | - | |
|  | **IV E** | - | - | - | | - | | - | | 2 (67%) | |
|  | | **Enhanced Reference Standard** | | | | | | | | | |
|  |  | I | II | | II E | | III | | IV | | IV E |
|  |  | N=2 | N=26 | | N=0 | | N=5 | | N=14 | | N=3 |
| **WB MRI**  **(Analysis 3)** | **I** | 2 (100%) | - | | - | | - | | - | | - |
|  | **II** | - | 26 (100%) | | - | | - | | - | | - |
|  | **II E** | - | - | | 0 (0%) | | - | | - | | 1 (33%) |
|  | **III** | - | - | | - | | 5 (100%) | | 1 (7%) | | - |
|  | **IV** | - | - | | - | | - | | 13 (92%) | | - |
|  | **IV E** | - | - | | - | | - | | - | | 2 (67%) |

**Analysis 1:** Comparison between WB-MRI and primary reference standard

**Analysis 2**: Comparison between WB-MRI and enhanced reference standard before removal of WB-MRI perceptual errors

**Analysis 3**: Comparison between WB-MRI and enhanced reference standard following removal of WB-MRI perceptual errors

**Supplemental Table 6:** List of nodal disease sites with discrepant interim treatment response between WB-MRI and enhanced reference standard.

| **Disease site** | **Reference standard response** | **WB-MRI response** | **Reason for discrepancy** |
| --- | --- | --- | --- |
| Cervical | CR | PRa | Percentage residual tumour 34% for WB-MRI and 19% for PET-CT |
| Inguinal | PRi | CR | Residual PET Positivity |
| Cervical | CR | PRa | Percentage residual tumour 26% for WB-MRI and 2.7% for PET-CT |
| Supraclavicular | CR | PRa | Percentage residual tumour 37% for WB-MRI and 3.8% for PET-CT |
| Mediastinal | PRa | CR | Percentage residual tumour 14% for WB-MRI and 40% for PET-CT |
| Supraclavicular | PRi | CR | Percentage residual tumour 6% for WB-MRI and 70% for PET-CT |
| Supraclavicular | PRa | CR | Percentage residual tumour 4.6% for WB-MRI and 38% for PET-CT |
| Mediastinal | PRi | CR | Residual PET positivity |
| Cervical | NC | PRi | Percentage residual tumour 53% for WB-MRI and 80% for PET-CT |
| Supraclavicular | NC | PRa | Percentage residual tumour 32.5% for WB-MRI and 84% for PET-CT |
| Cervical | PRa | CR | Percentage residual tumour 11% for WB-MRI and 42% for PET-CT |
| Liver hilar | PRi | CR | Percentage residual tumour 0 for WB-MRI and 57% for PET-CT |
| Iliac | PRa | CR | Percentage residual tumour 3.3% for WB-MRI and 25% for PET-CT |
| Mediastinal | PRa | CR | Percentage residual tumour 13% for WB-MRI and 28% for PET-CT |
| Supraclavicular | PRa | CR | Percentage residual tumour 6.4% for WB-MRI and 42% for PET-CT |
| Mediastinal | PRi | CR | Residual PET Positivity |
| Supraclavicular | PRi | CR | Residual PET Positivity |

**PRa:** Partial response adequate

**PRi:** Partial response inadequate

**CR:** Complete response

**NC:** No change

**WB-MRI:** Whole-body MRI

**PET-CT:** Positron emission tomography-computed tomography scan

**References:**

[1] Punwani S, Cheung KK, Skipper N, et al (2013) Dynamic contrast-enhanced MRI improves accuracy for detecting focal splenic involvement in children and adolescents with Hodgkin disease. Pediatr Radiol 43:941-949.

[2] Lassmann M, Biassoni L, Monsieurs M, Franzius C; EANM Dosimetry and Paediatrics Committees (2008) The new EANM paediatric dosage card: additional notes with respect to F-18. Eur J Nucl Med Mol Imaging 35:1666-1668.

[3] Kleis M, Daldrup-Link H, Matthay K, et al (2009) Diagnostic value of PET/CT for the staging and restaging of pediatrics tumors. Eur J Nucl Med Mol Imaging 36:23-36.

[4] Körholz D, Wallace H, Landman-Parker J (2006) EuroNet-Paediatric Hodgkin’s Lymphoma Group, First international Inter-Group Study for classical Hodgkin’s Lymphoma in Children and Adolescents, Radiotherapy Manual. https://www.skion.nl/workspace/uploads/euronet-phl-c1_workingcopy_inkl_amendm06_mw_2012-11-14_0.pdf

[5] Punwani S, Prakash V, Bainbridge A, et al (2010) Quantitative diffusion weighted MRI: A functional biomarker of nodal disease in Hodgkin’s lymphoma. Cancer Biomarker 7:249-259.

[6] Punwani S, Taylor SA, Bainbridge A, et al (2010) Pediatric and adolescent lymphoma: comparison of whole-body STIR half-Fourier RARE MR imaging with an enhanced PET/CT reference for initial staging. Radiology 255:182-190.
